# 5-Meth­oxy-1,3,4-thia­diazol-2(3*H*)-one

**DOI:** 10.1107/S160053681200178X

**Published:** 2012-01-21

**Authors:** Wei-Yi Zhang, Jie Liu, Yan-Ju Liu

**Affiliations:** a Department of Obstetrics and Gynecology, The First Affiliated Hospital of Henan University of Traditional Chinese, Medicine, Zhengzhou 450008, People’s Republic of China; b Department of Urology, Henan Provincial People’s Hospital, Zhengzhou 450003, People’s Republic of China; c Pharmacy College, Henan University of Traditional Chinese Medicine, Zhengzhou 450008, People’s Republic of China

## Abstract

The three mol­ecules in the asymmetric unit of the title compound, C_3_H_4_N_2_O_2_S, are connected *via* N—H⋯O hydrogen bonds, forming layers normal to [001]. The rings of the mol­ecules are approximately planar, with r.m.s. deviations of 0.0051 (1), 0.0044 (1) and 0.0111 (1) Å.

## Related literature

For background to the applications of the title compound, see: Collier (2004 ▶). For the synthesis, see: Zhu *et al.* (2011 ▶). For bond-length data, see: Allen *et al.* (1987 ▶).
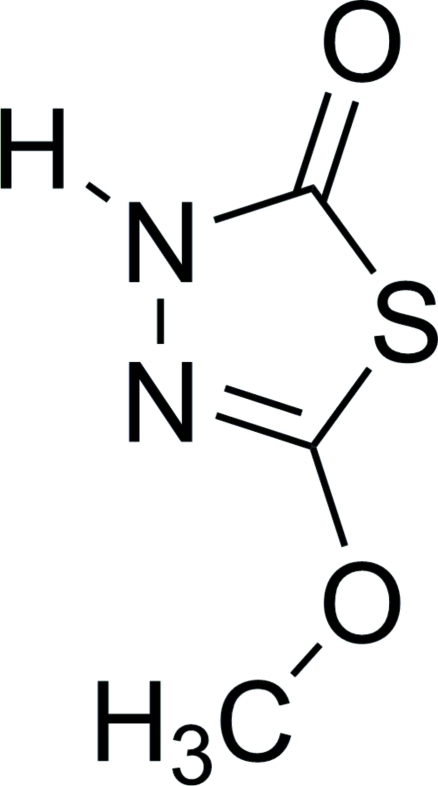


## Experimental

### 

#### Crystal data

C_3_H_4_N_2_O_2_S
*M* *_r_* = 132.16
Hexagonal, 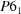

*a* = 11.9240 (17) Å
*c* = 20.111 (4) Å
*V* = 2476.3 (7) Å^3^
*Z* = 18
Mo *K*α radiation
μ = 0.49 mm^−1^
*T* = 293 K
0.30 × 0.20 × 0.20 mm

#### Data collection

Enraf–Nonius CAD-4 diffractometer
Absorption correction: ψ scan (North *et al.*, 1968 ▶) *T*_min_ = 0.867, *T*_max_ = 0.909
3492 measured reflections
3040 independent reflections
2206 reflections with *I* > 2σ(*I*)
*R*_int_ = 0.039
3 standard reflections every 200 reflections intensity decay: 1%

#### Refinement

*R*[*F* ^2^ > 2σ(*F* ^2^)] = 0.056
*wR*(*F* ^2^) = 0.133
*S* = 1.04
3040 reflections
217 parameters
1 restraint
H-atom parameters constrained
Δρ_max_ = 0.27 e Å^−3^
Δρ_min_ = −0.38 e Å^−3^
Absolute structure: Flack (1983 ▶), 1468 Friedel pairs
Flack parameter: −0.05 (14)

### 

Data collection: *CAD-4 Software* (Enraf–Nonius, 1985 ▶); cell refinement: *CAD-4 Software*; data reduction: *XCAD4* (Harms & Wocadlo, 1995 ▶); program(s) used to solve structure: *SHELXS97* (Sheldrick, 2008 ▶); program(s) used to refine structure: *SHELXL97* (Sheldrick, 2008 ▶); molecular graphics: *SHELXTL* (Sheldrick, 2008 ▶); software used to prepare material for publication: *SHELXTL*.

## Supplementary Material

Crystal structure: contains datablock(s) I, global. DOI: 10.1107/S160053681200178X/bq2331sup1.cif

Structure factors: contains datablock(s) I. DOI: 10.1107/S160053681200178X/bq2331Isup2.hkl

Supplementary material file. DOI: 10.1107/S160053681200178X/bq2331Isup3.cml

Additional supplementary materials:  crystallographic information; 3D view; checkCIF report

## Figures and Tables

**Table 1:** Hydrogen-bond geometry (Å, °)

*D*—H⋯*A*	*D*—H	H⋯*A*	*D*⋯*A*	*D*—H⋯*A*
N1—H1*A*⋯O3^i^	0.86	1.97	2.796 (7)	160
N3—H3*D*⋯O5	0.86	1.96	2.788 (6)	161
N5—H5*A*⋯O1^ii^	0.86	1.99	2.813 (8)	161

Symmetry codes: (i) 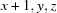; (ii) 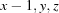.

**Fig. 1.**
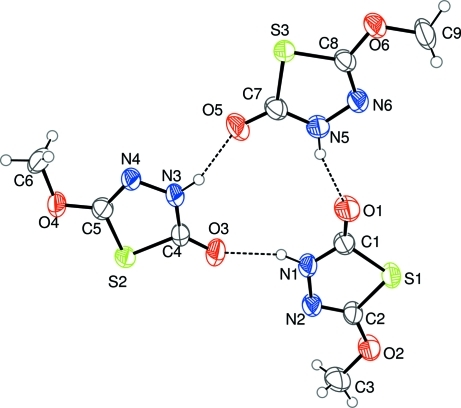
The molecular structure of (I), with the atom-numbering scheme. Displacement ellipsoids are drawn at the 50% probability level. Intermolecular H-bonds are shown with dashed lines.

**Fig. 2.**
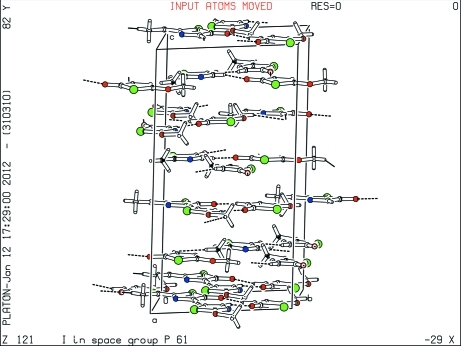
A packing diagram of (I) showing the stacked layers along c-axis.

## supplementary crystallographic information

### Comment

The tittle compound, 2-methoxythiazol-5(4*H*)-one is an important intermediate, which can be utilized to synthesize herbicide fluthiacet-ethyl (Collier, 2004). We report here the crystal structure of the title compound, (I).

The molecular structure of (I) is shown in Fig. 1. In the crystal structure, the asymmetric unit contains three molecules of 2-methoxythiazol-5(4*H*)-one and these molecules were connected together via N-H···O intermolecular hydrogen bonds forming stacking layers along c-axis (Fig. 2.). In the crystal structure, the rings are planar, with r.m.s. deviation of 0.06 (1) Å. The bond lengths and angles are within normal ranges (Allen *et al.*, 1987).

### Experimental

The title compound, (I) was prepared by a method reported in literature (Zhu *et al.*, 2011). The crystals were obtained by dissolving (I) (0.2 g) in methanol (50 ml) and evaporating the solvent slowly at room temperature for about 10 d.

### Refinement

All H atoms were positioned geometrically and constrained to ride on their parent atoms, with 0.86 Å for N—H, 0.96 Å for methyl H, respectively. The *U*_iso_(H) = *xU*_eq_(C), where *x* = 1.2 for N—H, and *x* = 1.5 for methyl H.

### Crystal data

**Table:**

C_3_H_4_N_2_O_2_S	*D*_x_ = 1.595 Mg m^−^^3^
*M**_r_* = 132.16	Mo *K*α radiation, λ = 0.71073 Å
Hexagonal, *P*6_1_	Cell parameters from 25 reflections
Hall symbol: P 61	θ = 9–13°
*a* = 11.9240 (17) Å	µ = 0.49 mm^−^^1^
*c* = 20.111 (4) Å	*T* = 293 K
*V* = 2476.3 (7) Å^3^	Block, colourless
*Z* = 18	0.30 × 0.20 × 0.20 mm
*F*(000) = 1224	

### Data collection

**Table:**

Enraf–Nonius CAD-4 diffractometer	2206 reflections with *I* > 2σ(*I*)
Radiation source: fine-focus sealed tube	*R*_int_ = 0.039
graphite	θ_max_ = 25.4°, θ_min_ = 2.0°
ω/2θ scans	*h* = 0→12
Absorption correction: ψ scan (North *et al.*, 1968)	*k* = 0→12
*T*_min_ = 0.867, *T*_max_ = 0.909	*l* = −24→24
3492 measured reflections	3 standard reflections every 200 reflections
3040 independent reflections	intensity decay: 1%

### Refinement

**Table:**

Refinement on *F*^2^	Hydrogen site location: inferred from neighbouring sites
Least-squares matrix: full	H-atom parameters constrained
*R*[*F*^2^ > 2σ(*F*^2^)] = 0.056	*w* = 1/[σ^2^(*F*_o_^2^) + (0.0696*P*)^2^ + 0.1426*P*] where *P* = (*F*_o_^2^ + 2*F*_c_^2^)/3
*wR*(*F*^2^) = 0.133	(Δ/σ)_max_ < 0.001
*S* = 1.04	Δρ_max_ = 0.27 e Å^−^^3^
3040 reflections	Δρ_min_ = −0.38 e Å^−^^3^
217 parameters	Extinction correction: *SHELXL97* (Sheldrick, 2008)
1 restraint	Extinction coefficient: 0.0009 (2)
Primary atom site location: structure-invariant direct methods	Absolute structure: Flack (1983), 1468 Friedel pairs
Secondary atom site location: difference Fourier map	Flack parameter: −0.05 (14)

### Special details

**Table:**

Geometry. All e.s.d.'s (except the e.s.d. in the dihedral angle between two l.s. planes) are estimated using the full covariance matrix. The cell e.s.d.'s are taken into account individually in the estimation of e.s.d.'s in distances, angles and torsion angles; correlations between e.s.d.'s in cell parameters are only used when they are defined by crystal symmetry. An approximate (isotropic) treatment of cell e.s.d.'s is used for estimating e.s.d.'s involving l.s. planes.
Refinement. Refinement of *F*^2^ against ALL reflections. The weighted *R*-factor *wR* and goodness of fit *S* are based on *F*^2^, conventional *R*-factors *R* are based on *F*, with *F* set to zero for negative *F*^2^. The threshold expression of *F*^2^ > σ(*F*^2^) is used only for calculating *R*-factors(gt) *etc*. and is not relevant to the choice of reflections for refinement. *R*-factors based on *F*^2^ are statistically about twice as large as those based on *F*, and *R*- factors based on ALL data will be even larger.

### Fractional atomic coordinates and isotropic or equivalent isotropic displacement parameters (Å^2^)

**Table:**

	*x*	*y*	*z*	*U*_iso_*/*U*_eq_	
S1	0.82079 (12)	0.67453 (12)	0.52166 (9)	0.0559 (4)	
N1	0.8459 (4)	0.4789 (4)	0.5285 (3)	0.0500 (12)	
H1A	0.8783	0.4288	0.5317	0.060*	
C1	0.9238 (5)	0.6063 (5)	0.5255 (3)	0.0473 (14)	
O1	1.0407 (3)	0.6672 (4)	0.5228 (3)	0.0709 (14)	
N2	0.7133 (4)	0.4265 (4)	0.5265 (3)	0.0487 (12)	
O2	0.5724 (3)	0.5049 (3)	0.5201 (2)	0.0638 (12)	
C2	0.6895 (5)	0.5184 (5)	0.5232 (3)	0.0440 (13)	
C3	0.4662 (5)	0.3737 (6)	0.5148 (4)	0.071 (2)	
H3A	0.3860	0.3742	0.5130	0.107*	
H3B	0.4659	0.3248	0.5528	0.107*	
H3C	0.4759	0.3348	0.4751	0.107*	
S2	−0.10862 (12)	0.04825 (12)	0.51466 (7)	0.0452 (4)	
O3	−0.1023 (4)	0.2749 (4)	0.5220 (3)	0.0646 (12)	
O4	0.0620 (3)	−0.0301 (3)	0.5161 (2)	0.0564 (11)	
N3	0.0884 (4)	0.2708 (4)	0.5190 (3)	0.0497 (12)	
H3D	0.1382	0.3535	0.5191	0.060*	
N4	0.1407 (4)	0.1911 (4)	0.5184 (3)	0.0443 (11)	
C4	−0.0410 (5)	0.2188 (5)	0.5194 (3)	0.0431 (13)	
C5	0.0477 (4)	0.0735 (5)	0.5165 (3)	0.0429 (12)	
C6	0.1950 (5)	−0.0021 (6)	0.5226 (4)	0.0588 (16)	
H6A	0.1968	−0.0817	0.5216	0.088*	
H6B	0.2302	0.0416	0.5640	0.088*	
H6C	0.2456	0.0521	0.4864	0.088*	
S3	0.52008 (12)	0.74536 (12)	0.53340 (7)	0.0513 (4)	
O5	0.2932 (4)	0.5245 (3)	0.5299 (3)	0.0760 (14)	
O6	0.5965 (3)	0.9929 (3)	0.5329 (2)	0.0622 (12)	
N5	0.2976 (4)	0.7178 (4)	0.5261 (3)	0.0519 (12)	
H5A	0.2149	0.6839	0.5233	0.062*	
N6	0.3771 (4)	0.8501 (4)	0.5268 (3)	0.0500 (11)	
C7	0.3507 (5)	0.6438 (5)	0.5296 (3)	0.0486 (14)	
C8	0.4939 (4)	0.8748 (5)	0.5309 (3)	0.0447 (13)	
C9	0.5707 (6)	1.0982 (5)	0.5339 (5)	0.074 (2)	
H9A	0.6511	1.1787	0.5356	0.111*	
H9B	0.5198	1.0907	0.5725	0.111*	
H9C	0.5240	1.0955	0.4945	0.111*	

### Atomic displacement parameters (Å^2^)

**Table:**

	*U*^11^	*U*^22^	*U*^33^	*U*^12^	*U*^13^	*U*^23^
S1	0.0353 (7)	0.0322 (7)	0.1008 (12)	0.0171 (6)	0.0044 (9)	0.0039 (8)
N1	0.038 (2)	0.035 (2)	0.082 (4)	0.023 (2)	−0.001 (3)	−0.003 (3)
C1	0.034 (3)	0.039 (3)	0.069 (4)	0.019 (3)	0.003 (3)	−0.004 (3)
O1	0.033 (2)	0.050 (2)	0.125 (4)	0.0174 (18)	0.004 (3)	−0.004 (3)
N2	0.035 (2)	0.035 (2)	0.076 (4)	0.0171 (19)	0.001 (2)	0.000 (3)
O2	0.0308 (18)	0.043 (2)	0.117 (4)	0.0178 (16)	0.004 (2)	−0.003 (3)
C2	0.031 (2)	0.033 (3)	0.066 (4)	0.014 (2)	0.008 (3)	0.007 (3)
C3	0.036 (3)	0.053 (4)	0.115 (7)	0.015 (3)	−0.006 (4)	−0.011 (4)
S2	0.0315 (6)	0.0334 (6)	0.0664 (9)	0.0132 (5)	−0.0002 (6)	−0.0008 (6)
O3	0.059 (3)	0.047 (2)	0.101 (3)	0.036 (2)	−0.005 (3)	−0.004 (2)
O4	0.045 (2)	0.0305 (19)	0.096 (3)	0.0208 (16)	−0.003 (2)	−0.001 (2)
N3	0.042 (2)	0.025 (2)	0.079 (4)	0.0145 (18)	−0.006 (3)	0.001 (2)
N4	0.035 (2)	0.029 (2)	0.066 (3)	0.0139 (17)	0.000 (3)	0.004 (2)
C4	0.043 (3)	0.031 (2)	0.057 (4)	0.020 (2)	−0.002 (3)	0.000 (3)
C5	0.038 (3)	0.037 (3)	0.052 (3)	0.017 (2)	0.002 (3)	0.003 (3)
C6	0.044 (3)	0.055 (3)	0.088 (5)	0.032 (3)	0.001 (4)	0.016 (4)
S3	0.0317 (7)	0.0351 (7)	0.0863 (12)	0.0160 (6)	−0.0036 (7)	−0.0017 (7)
O5	0.045 (2)	0.029 (2)	0.139 (4)	0.0071 (19)	−0.003 (3)	−0.001 (3)
O6	0.036 (2)	0.0323 (19)	0.110 (4)	0.0112 (17)	0.001 (2)	0.001 (2)
N5	0.029 (2)	0.041 (2)	0.084 (4)	0.016 (2)	0.004 (3)	−0.001 (3)
N6	0.036 (2)	0.034 (2)	0.078 (3)	0.0164 (19)	0.003 (3)	0.001 (2)
C7	0.036 (3)	0.037 (3)	0.067 (4)	0.014 (2)	0.003 (3)	−0.005 (3)
C8	0.033 (3)	0.032 (3)	0.064 (4)	0.012 (2)	0.003 (3)	−0.002 (3)
C9	0.059 (4)	0.027 (3)	0.127 (6)	0.016 (3)	0.009 (4)	0.004 (4)

### Geometric parameters (Å, °)

**Table:**

S1—C2	1.733 (5)	N3—N4	1.373 (5)
S1—C1	1.782 (5)	N3—H3D	0.8600
N1—C1	1.328 (6)	N4—C5	1.282 (6)
N1—N2	1.380 (5)	C6—H6A	0.9600
N1—H1A	0.8600	C6—H6B	0.9600
C1—O1	1.209 (6)	C6—H6C	0.9600
N2—C2	1.264 (6)	S3—C8	1.721 (5)
O2—C2	1.324 (6)	S3—C7	1.762 (6)
O2—C3	1.443 (7)	O5—C7	1.232 (6)
C3—H3A	0.9600	O6—C8	1.326 (6)
C3—H3B	0.9600	O6—C9	1.434 (7)
C3—H3C	0.9600	N5—C7	1.321 (6)
S2—C5	1.734 (5)	N5—N6	1.375 (6)
S2—C4	1.777 (5)	N5—H5A	0.8600
O3—C4	1.214 (5)	N6—C8	1.274 (6)
O4—C5	1.328 (5)	C9—H9A	0.9600
O4—C6	1.454 (6)	C9—H9B	0.9600
N3—C4	1.345 (6)	C9—H9C	0.9600
			
C2—S1—C1	88.1 (2)	N4—C5—O4	125.1 (4)
C1—N1—N2	120.3 (4)	N4—C5—S2	117.2 (4)
C1—N1—H1A	119.9	O4—C5—S2	117.7 (3)
N2—N1—H1A	119.9	O4—C6—H6A	109.5
O1—C1—N1	128.8 (5)	O4—C6—H6B	109.5
O1—C1—S1	125.1 (4)	H6A—C6—H6B	109.5
N1—C1—S1	106.1 (4)	O4—C6—H6C	109.5
C2—N2—N1	108.2 (4)	H6A—C6—H6C	109.5
C2—O2—C3	115.9 (4)	H6B—C6—H6C	109.5
N2—C2—O2	125.2 (4)	C8—S3—C7	87.5 (2)
N2—C2—S1	117.3 (4)	C8—O6—C9	116.3 (4)
O2—C2—S1	117.5 (4)	C7—N5—N6	118.7 (5)
O2—C3—H3A	109.5	C7—N5—H5A	120.7
O2—C3—H3B	109.5	N6—N5—H5A	120.7
H3A—C3—H3B	109.5	C8—N6—N5	108.2 (4)
O2—C3—H3C	109.5	O5—C7—N5	126.5 (5)
H3A—C3—H3C	109.5	O5—C7—S3	125.4 (4)
H3B—C3—H3C	109.5	N5—C7—S3	108.1 (4)
C5—S2—C4	88.1 (2)	N6—C8—O6	124.6 (4)
C5—O4—C6	114.7 (4)	N6—C8—S3	117.5 (4)
C4—N3—N4	119.6 (4)	O6—C8—S3	117.8 (3)
C4—N3—H3D	120.2	O6—C9—H9A	109.5
N4—N3—H3D	120.2	O6—C9—H9B	109.5
C5—N4—N3	108.3 (4)	H9A—C9—H9B	109.5
O3—C4—N3	127.9 (5)	O6—C9—H9C	109.5
O3—C4—S2	125.4 (4)	H9A—C9—H9C	109.5
N3—C4—S2	106.7 (3)	H9B—C9—H9C	109.5
			
N2—N1—C1—O1	−175.3 (7)	N3—N4—C5—S2	0.5 (7)
N2—N1—C1—S1	1.7 (7)	C6—O4—C5—N4	4.0 (10)
C2—S1—C1—O1	176.1 (6)	C6—O4—C5—S2	−175.8 (5)
C2—S1—C1—N1	−1.0 (5)	C4—S2—C5—N4	−1.8 (5)
C1—N1—N2—C2	−1.5 (8)	C4—S2—C5—O4	178.0 (5)
N1—N2—C2—O2	179.6 (6)	C7—N5—N6—C8	0.2 (9)
N1—N2—C2—S1	0.5 (8)	N6—N5—C7—O5	179.6 (7)
C3—O2—C2—N2	−5.2 (10)	N6—N5—C7—S3	−0.8 (7)
C3—O2—C2—S1	174.0 (5)	C8—S3—C7—O5	−179.5 (7)
C1—S1—C2—N2	0.3 (6)	C8—S3—C7—N5	0.9 (5)
C1—S1—C2—O2	−178.9 (5)	N5—N6—C8—O6	−179.9 (6)
C4—N3—N4—C5	1.7 (8)	N5—N6—C8—S3	0.6 (8)
N4—N3—C4—O3	177.5 (7)	C9—O6—C8—N6	3.4 (11)
N4—N3—C4—S2	−2.9 (7)	C9—O6—C8—S3	−177.1 (6)
C5—S2—C4—O3	−178.0 (6)	C7—S3—C8—N6	−0.9 (6)
C5—S2—C4—N3	2.4 (5)	C7—S3—C8—O6	179.6 (6)
N3—N4—C5—O4	−179.3 (6)		

### Hydrogen-bond geometry (Å, °)

**Table:**

*D*—H···*A*	*D*—H	H···*A*	*D*···*A*	*D*—H···*A*
N1—H1A···O3^i^	0.86	1.97	2.796 (7)	160
N3—H3D···O5	0.86	1.96	2.788 (6)	161
N5—H5A···O1^ii^	0.86	1.99	2.813 (8)	161

Symmetry codes: (i) *x*+1, *y*, *z*; (ii) *x*−1, *y*, *z*.
